# Learning from real-world conditions to advance green care for mental health equity in the Global North: protocol for a transdisciplinary multi-scalar project

**DOI:** 10.1136/bmjopen-2025-114034

**Published:** 2026-07-29

**Authors:** Helen V S Cole, Silvio Caputo, Kathrin Specht, Renata Giedych, Noriko Otsuka, Penny A Cook, Beata Gawryszewska, Juan V Luciano, Agnieszka Olszewska-Guizzo, Marcus Hedblom, Rebecca Houston-Read, Katelyn McVay, Konrad Neuberger, Halina Sienkiewicz-Jarosz, Albert Feliu-Soler, Paula de Prado-Bert, Michael Hardman, Michelle Louise Howarth, Rachel Bragg, Antonella Crichigno, Margarita Triguero-Mas

**Affiliations:** 1 Pediatrics, Obstetrics, Gynecology, Preventive Medicine and Public Health, Universitat Autònoma de Barcelona, Barcelona, Spain; 2 Serra Húnter Programme, Generalitat de Catalunya, Barcelona, Spain; 3 Barcelona Lab for Urban Environmental Justice and Sustainability, ICTA-UAB, Universitat Autònoma de Barcelona, Bellaterra, Spain; 4 Kent School of Architecture and Planning, University of Kent, Canterbury, UK; 5 Research Institute for Regional and Urban Development, Dortmund, Germany; 6 Institute of Environmental Engineering, Department of Landscape Architecture, Warsaw University of Life Sciences, Warsaw, Poland; 7 College of Health & Social Care, School of Health Sciences, Salford, UK; 8 Universitat Autònoma de Barcelona, Barcelona, Spain; 9 Parc Sanitari Sant Joan de Deu, Sant Boi de Llobregat, Spain; 10 Epidemiology and Public Health, CIBER, Madrid, Spain; 11 NeuroLandscape NGO, Warsaw, Poland; 12 Department of Urban and Rural Development, Swedish University of Agricultural Sciences, Uppsala, Sweden; 13 Department of Environmental Health Sciences, Emory University Rollins School of Public Health, Atlanta, Georgia, USA; 14 Gesellschaft für Gartenbau und Therapie GGuT e.V, Hueckeswagen, Germany; 15 Institute of Psychiatry and Neurology in Warsaw, Warsaw, Poland; 16 Maria Sklodowska-Curie Medical University, Warsaw, Poland; 17 Environmental Research & Innovation Centre, University of Salford, Salford, UK; 18 Edge Hill University, Ormskirk, UK; 19 Social Farms and Gardens, Bristol, UK; 20 Old Continent, Brussels, Belgium; 21 Barcelona InTerdisciplinary research group on plAnetary heaLth (BITAL), Universitat Oberta de Catalunya Estudis de Ciencies de la Salut, Barcelona, Spain; 22 ISGlobal, Barcelona, Spain; 23 Universitat Pompeu Fabra, Barcelona, Spain

**Keywords:** MENTAL HEALTH, Research Design, Health Equity, EPIDEMIOLOGIC STUDIES, Health Services

## Abstract

**Introduction:**

There is increasing evidence that green care (from nature-in-everyday life and nature-based health promotion to nature-based therapy) can promote mental health and well-being. However, there is contradictory evidence and few large-scale international studies focused on health inequities. The GreenME project aims to identify ways in which effective green care can be scaled-up to improve adult mental health and well-being equity while contributing to multiple socio-ecological co-benefits across the Global North.

**Methods and analysis:**

GreenME’s approach is threefold: (i) to diagnose the current status of green care in project consortium countries through a grey literature review and stakeholder interviews, (ii) to increase scientific evidence on the relationship between green care and mental health and well-being equity using modified randomised controlled trials and a population-level socioecological cross-sectional survey study and (iii) to empower green care actors by co-creating a set of tools with consortium countries’ participating stakeholders. GreenME will: (a) produce a catalogue of identified successful green care models and best practices; (b) design a robust but adaptable protocol for testing the pathways, effectiveness and cost-effectiveness of nature-based therapies; (c) identify the mental health equity impact of green care and (e) co-create solutions for national and EU-level policies and develop online resources for nature-based therapy providers. GreenME is conceptualised and designed to increase the use of green care and its integration within a multi-scalar green care framework which could ultimately promote just and sustainable healthy communities.

**Ethics and dissemination:**

Ethical approval has been obtained individually from each partner/site (UAB CERec 6594, 6851, UAB-CERec180, UAB-CERec178 and CEEAH 7269; UOC CE23-PR29, CE240PR07, CE24-PR59, CE24-PR55; l'Hospital de Mataró CEIm 11/2; CEIm Fundació Sant Joan de Déu PIC-82-25; Alma Mater Stadiorum Universita di Bologna, Proj nos. 0157655, 0075975; ILS, no number ‘Survey in Workpackage 2’, no number ‘Workshops in Workpackage 5’; Swedish Ethics Review Committee, 2024–2810-01, 2025–0195401; University of Kent, CREAG064-04-024; University of Salford Ethics Administration, Ref 0189, 4552; Institute of Psychiatry and Neurology Bioethics Committee, no number; Committees on Ethics and Scientific Research, Res no 32/RKF/2023U). All study results will be disseminated through peer-reviewed publications, scientific conferences and via reports and workshops for stakeholders at the regional (European Union), national and local levels.

**Trial registration number:**

ISRCTN13105773, ISRCTN71485431, ISRCTN11841855, ISRCTN14970007 and ISRCTN15381820.

**STRENGTHS AND LIMITATIONS OF THIS STUDY**

- This study adds a health equity framing across its multiple design elements.
- We will evaluate existing nature-based therapy interventions, capturing those interventions which have already survived in the real world while gathering missing evidence on their mechanisms and cost-effectiveness.
- The study gathers population-level mental health and equity data and exposures to green and blue spaces across study sites in seven countries.
- The trade-offs of evaluating existing interventions may reduce the robustness of the study design.

## Introduction

 At least 15% (95% CI 16.8% to 16.8%) of the population globally lives with mental ill-health.^1^ This is likely to be an underestimate due to widespread stigma towards mental health problems and subsequent underreporting. It is vital to address mental health through an equity lens, which considers the ability of all people to achieve their best possible state of mental health without unfair, avoidable or remediable differences between groups.^2^ Historical, cultural, political and sociodemographic factors impact how mental health problems are unevenly distributed, perceived and addressed.^3^

Mental health problems are the third leading cause of overall disease burden in Europe (including the UK), affecting approximately 150 million people.^4^ However, individuals who are members of marginalised groups (ie, those who have fewer opportunities compared with other social groups and may experience a higher risk of poverty, social exclusion, discrimination and/or violence than the general population due to social, cultural, economic and political conditions or a combination of these conditions; see table 1) experience a disproportionate burden of these problems,^5^ partly due to unequal social and environmental conditions.^6^ For example, rural populations may have less access to mental health services, while those who live in urban areas may face environmental stressors such as air pollution or lack of green space.^7–9^ Moreover, each country and region has context-specific public health challenges.^10^ Addressing the growing burden of mental health problems across the Global North thus requires multifaceted solutions that take country context and the intersectionality of privilege and marginalisation into account.^3^

**Table 1:** Key GreenME terminology

Definitions were drafted and refined by the GreenME terminology working group with consultation from GreenME consortium members	
Nature	Within the context of the GreenME project, **nature** is defined as the natural world, including both its living and non-living aspects such as plants, animals, rocks and water, as well as the processes and phenomena that produce, reproduce and occur within it, such as plant growth and wind and tides. This understanding of nature does not exclude human influence and presence, thus including ‘man-made’ areas (eg, gardens, parks, hiking trails, etc.) as aspects of nature.
Green care	GreenME defines **green care** as a way of conceptualising different ways of engaging with nature that can improve human health and well-being. It is framed within a three-level model from nature-in-everyday life and nature-based health promotion to nature-based therapy.
Nature-based therapy (NBT)	**Nature-based therapy** is a therapeutic intervention directed by a trained facilitator in an indoor or an outdoor setting that includes exposure to and interaction with nature to enhance individuals’ physical and mental health and well-being. Examples of NBTs: participating in a structured horticulture therapy programme led by a trained professional designed for people with high levels of anxiety or participating in a forest therapy programme led by a psychologist and designed for people suffering from prolonged grief.
Nature-based health promotion (NBP)	**Nature-based health promotion** uses organised activities to support people to engage with nature, leading to improvements in health and well-being. These initiatives offer nature-based activities within an organised structure without specific therapeutic aims. Examples of NBP activities: walking in nature as part of walking groups promoted by the local health department or participating in an organised community gardening initiative.
Nature-in-everyday life (NEL)	**Nature-in-everyday life** provides opportunities for engagement with nature as part of people’s everyday lives, leading to improvements in health and well-being. Examples of NEL: spending time in a park near your home or growing vegetables and flowers in your home garden is passive engagement with nature.
Marginalised groups	Population groups that have fewer opportunities than other groups in society and may experience a higher risk of poverty, social exclusion, discrimination and/or violence than the general population due to social, cultural, economic and political conditions or a combination of these conditions. Some examples include, but are not limited to, racial or ethnic minorities, migrants, people living with disabilities, older people experiencing isolation, people experiencing unemployment and people belonging to the LGBTIQ+ (Lesbian, Gay, Bisexual, Trans, Intersex, and Queer and others) community.
Environmental justice	GreenME views **environmental justice** as fair and equal access to and benefit from environmental resources such as green care, regardless of one’s personal characteristics.

NBT, nature-based therapy.

It is well established that exposure to green, such as parks or gardens, or blue spaces, such as waterfronts or natural areas with prominent water features, is linked to better mental health, including lower levels of stress, depression and anxiety.^11^ For instance, one recent study indicates that transforming a third of the Barcelona city streets into green corridors could prevent between 8 and 14% of cases of various mental health problems, including more than 30 000 cases of self-perceived poor mental health yearly.^12^ Beyond everyday exposure to nature, growing evidence also supports the effectiveness of more targeted and tailored nature-based interventions for improving mental health. For example, in a study with 36 middle-aged women in South Korea, it was found that 12 sessions of horticulture therapy significantly improved depressive, cognitive and anxiety outcomes compared with controls.^13^ Similarly, a systematic review of nature-based health promotion (NBP) programmes (ie, green exercise and spending time ‘savouring’ nature) for workers reported significant positive effects on mental health and well-being in most studies.^14^

Increasingly, inequities in the nature-health relationship have been acknowledged. Environmental psychology and epidemiology studies suggest that marginalised individuals may experience relatively greater benefits from everyday exposure to nature, perhaps due to the comparative lack of access to other healthful resources or having worse initial health outcomes and thus greater potential for improved outcomes.^15–17^ Conversely, social science research highlights that green and blue spaces closer to where marginalised individuals live are often of lower quality,^18^ and that these environments can be perceived sometimes as unsafe due to personal or community-linked histories of hostility or violence in these spaces.^19–21^ Moreover, recent studies have linked the improvement of green and blue spaces with sustainability gentrification processes, which may further exclude marginalised groups from benefiting.^22–24^ NBP and nature-based therapy (NBT) activities may also be less accessible to these groups due to time, cost or caregiving constraints. Finally, the limited integration of nature-based approaches into healthcare systems means that those who might benefit most often have the least access.

Our project, Advancing Greencare in Europe, an integrated multi-scalar approach for the expansion of nature-based therapies to improve mental health equity (GreenME) is grounded in the assumption that the role of green and blue spaces in improving mental health equity can be better approached through a green care framework that highlights the interconnectedness of three levels: nature-in-everyday life (NEL), NBP and NBT. Our understanding of green care purposefully diverges from published definitions, which often restrict it to individualised, facilitated interventions^25 26^ or overlook the role that everyday contact with nature plays in supporting mental health and wellbeing.^27–29^ The aim of GreenME is to conceptualise all three levels of green care as components of essential health infrastructure, identifying context-specific challenges and opportunities and providing robust evidence to support political and financial investment in effective and equitable nature-based care, with additional social and ecological co-benefits.^30^

## Methods

Over a period of 4 years (Sept 2023—August 2027), GreenME employs a three-phase process ((i) diagnosis, (ii) increasing the scientific evidence and (iii) empowering green care actors) to better understand the ways in which effective green care strategies might be scaled up to improve adult mental health and well-being equity across Europe and beyond. Throughout, GreenME prioritises learning from existing green care interventions. It uses a transdisciplinary approach—consisting of co-creation methodologies and the incorporation of both academic and non-academic stakeholders in the design of research protocols—and is informed by a health equity and environmental justice framework. Half of the consortium partners are non-academic entities, including NBT providers, green care associations or local governments. The project also benefits from a multi-disciplinary advisory board which includes researchers, practitioners and policy-makers.

### Geographic scope

**Figure 1:**
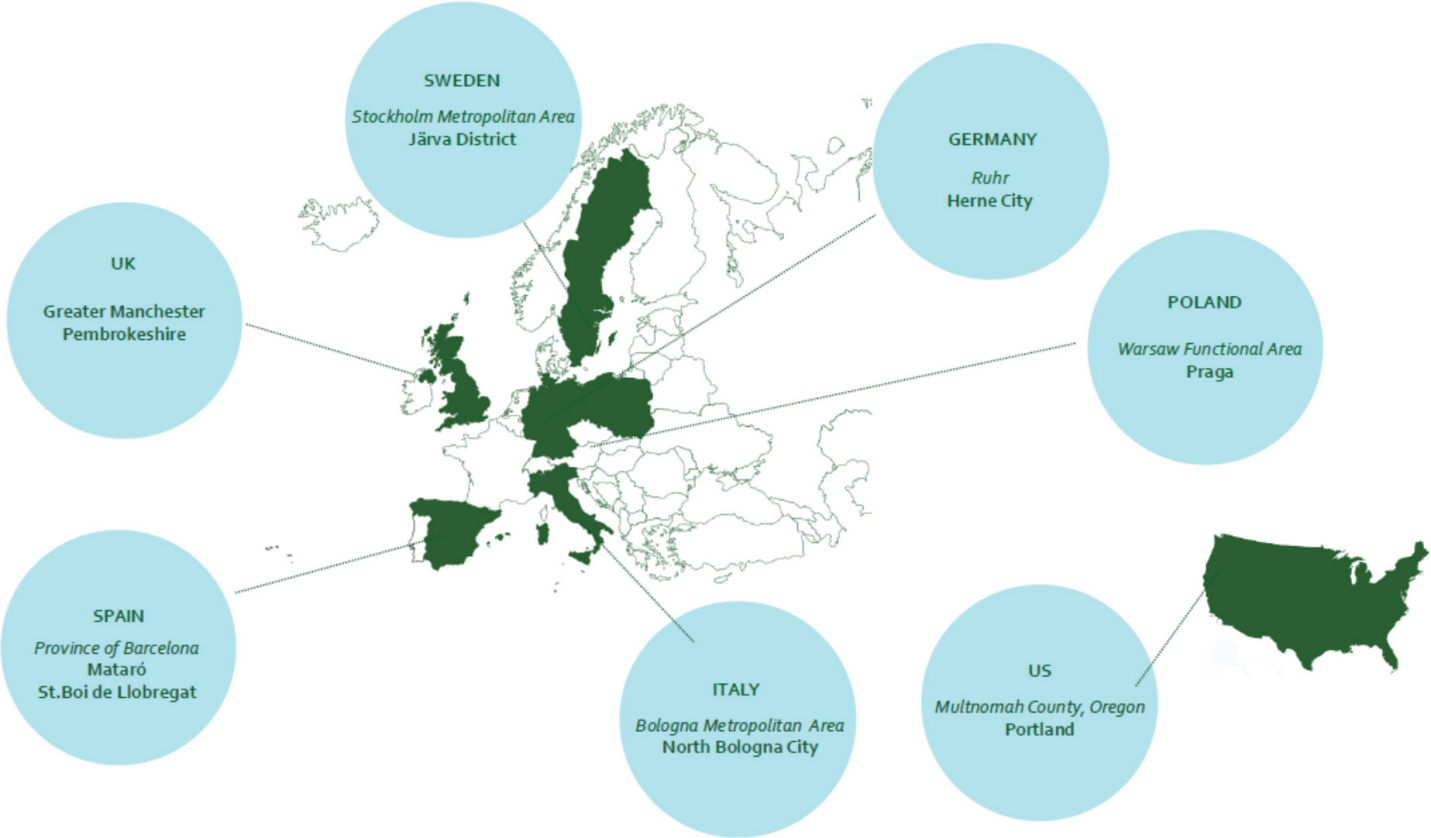
GreenME study areas analysed in Phase 1 (in italics) and sub-areas analysed in Phase 2 (in bold).

GreenME is being carried out in seven countries (Germany, Italy, Poland, Spain, Sweden, the UK and the US) with a variety of green care delivery partners and with healthcare structures. In each country, specific study areas represent regions with (i) diverse natural characteristics and levels of urbanicity and (ii) large populations of marginalised groups (as defined above). The specific study areas were used for the diagnosis phase of the project. In each of the specific study areas (see figure 1), sub-areas were selected for evaluating the role of NEL, NBP and NBT. In each sub-area, more than 50% of the population is socioeconomically deprived (ie, within the fourth and fifth quintiles of material and socioeconomic deprivation indexes available in each study area). In four of the seven countries where NBTs have a greater presence, we also evaluate existing NBTs. The evaluated NBTs (see table 2) are in the sub-areas.

**Table 2:** NBT cases in those countries where trials are being conducted

Name	Target population	Type of therapy
Italy		
SalusSpace	Adults living or working in Bologna city	Horticultural
BattimareSpace	Adults living or working in Bologna city	Horticultural
Spain		
CSMaresme	Adults that attend a mental health centre that is part of a day hospital	Horticultural
PSStJoanDéu	Adults who are grieving a loss and are participating in a grief support group in an institution that is part of the healthcare system	Forest bathing
Sweden		
Shrinyin-yoku	Adults who attend mental health centres in the Stockholm area	Forest bathing
United Kingdom		
Tonic	Adults with mental health problems referred to the programme by doctors, community mental health teams other charities or self-referred	Surf
Northern Roots	Adults with mental health problems referred to the programme by doctors, community mental health teams, other charities or themselves	Horticultural

### Phase 1: diagnosis of green care status

Phase 1 (data collection completed in August 2024) aimed to diagnose the green care status in each of the study areas. Using a holistic approach with an environmental justice lens, informed by frameworks such as ‘health in all policies’,^31^ we first characterised a range of relevant actors and mapped key stakeholders at different scales of green care according to extent of influence and level of expertise. We then (1) conducted semi-structured interviews with key actors in each study area (see online supplemental annex 1 for the interview guide employed, which was also translated into the appropriate local language) and (2) conducted an analysis of grey literature. The grey literature items were sought by study teams in each country by searching the official websites of the stakeholders identified during the first phase (key actor mapping) of the research. The search covered documents, databases, information channels, guidelines or other resources that have been developed at the national, regional or local level and have been (1) prepared by or commissioned by public authorities/institutions or (2) recommended by public authorities/institutions. In the absence of such documents, grey literature items prepared by professional and scientific associations and non-governmental organisations of major importance dealing with (1) health and well-being or (2) green-blue spaces were taken into consideration. The aim was to examine a broad range of laws, planning and programming documents, guidelines and schemes that impact the existence of green and blue spaces and promote one or more of the three levels of green care. The grey literature items analysed related to specific fields which are relevant to green care, such as land-use planning, nature conservation, environmental protection, forestry, agriculture, land management, climate change, healthcare, social welfare, education, public statistics and public finances. The READ approach^32^ was used to analyse the grey literature documents to assess the complementarity of health and green and blue space policies, as well as other policies relevant to green care development.

Together, the interviews and grey literature analysis examined the state of implementation of the three levels of green care in each partner country and the challenges of scaling up existing successful initiatives in different health systems, geographical areas and management regimes, considering the barriers and challenges faced in each. In addition, using these two data sources, we explored the level of integration between different policies in terms of health, green and blue infrastructure, social equity and environmental justice from the perspective of local and regional governance. Results of Phase 1 will further inform Phase 2 and 3 analyses.

### Phase 2: increase scientific evidence for green care

Phase 2 consists of two sub-studies focused on (1) NBTs targeted for specific populations and (2) those aspects of green care (NBP and NEL) which have implications for the mental health (equity) of the general population.

#### Effectiveness, causal pathways and cost-effectiveness of NBT programmes

We first developed a theory of change to clarify how and through which causal pathways nature-based therapies are understood to improve adult mental health and well-being (equity), informed by the published literature and by an eDelphi consensus-making process to capture the diverse expertise of the consortium and sister project members. This process consisted of two iterative surveys—the first asking open-ended questions to respondents relating to the constructs commonly included in a theory of change (ie, inputs—resources to implement the programme or project, activities—actions taken using the inputs, outputs—immediate, tangible service or products delivered by the programme or project, outcomes—measurable short- to medium-term effects that occur due to the outputs and expected impact—long-term change that the programme or project aims to achieve).^33^ In the second survey, the responses to the first survey were processed into a series of unique statements. Respondents were then asked to rank each statement in terms of relevance and importance, and those statements which were agreed on as both important and relevant were then included in a final theory of change by members of the research team.

Consortium members, including NBT practitioners and academic researchers, then iteratively co-created a common general protocol to evaluate seven existing NBTs (see table 2 above). We started with a study design as close as possible to the ‘gold standard’ randomised-controlled trial, establishing standardised practices and procedures for data collection, processing and analysis; randomisation of participants and measurement tools which will be used across all cases. All seven trials will record: (i) characteristics of each NBT case, (ii) pre- and post-study contact with NEL and NBP, (iii) mental health and well-being, (iv) information on direct (ie, medical visits, inpatient stays, lab tests, drug consumption) and indirect medical costs (days on sick leave, reduced productivity) and (v) participant demographics; and qualitative data which will inform our understanding of (i) the mechanisms linking NBT to mental health outcomes and (ii) the differential mental health benefits linked to intersectional axes of power and privilege. The general protocol was then tailored for each specific NBT case, adapting those elements necessary to consider their unique possibilities, characteristics and challenges.

Finally, the adapted, tailored protocol is being implemented to evaluate each case. This sub-study is designed to achieve sufficient statistical power to explore the relationships between exposure to NBT and mental health outcomes and cost-effectiveness for each intervention. Additionally, a meta-analysis of data from all sites will be conducted. Findings will be instrumental in enabling the mainstreaming of NBT on a wider scale and provide crucial data on interventions and their effects.

#### Mental health and well-being effects of NBP and NEL

To evaluate the effect of NEL and NBP on mental health and well-being (equity), we surveyed population samples in each sub-area, auditing natural spaces and using satellite imagery and GIS to identify space types and accessibility. This allows us to assess: (i) the quality and provision of green and blue spaces and the organised activities available for experiencing NEL and NBP; (ii) the impact of nature exposure on mental health and well-being (in)equity and (iii) the perceived qualities of nature, barriers to regular use and awareness of benefits that interaction with nature can generate.

The survey was co-designed by consortium partners so that it is adapted to the diverse local geographical and cultural contexts among study areas. It included questions about (i) duration of contact with nature, (ii) mental health and well-being status, (iii) connectedness with nature and (iv) the socio-demographic profile of respondents. The survey also included an interactive mapping component to assess actual exposure to NEL, including information about the types of activities done in nature and time spent in each space. To assess the quality and provision of a subset of natural spaces in each of the sub-areas, we designed and validated an audit toolkit that measures space quality (eg, access, amenities, biodiversity, landscape aesthetics,^34^ etc.) and environmental justice indicators.^35^ The audit was conducted in 50 selected areas in each sub-area, prioritising those reported in the interactive mapping component of the survey. Spatial and statistical methods are being used to assess associations between factors related to the mental health and well-being of respondents and their use of green and blue space, with results clustered according to socio-demographic profiles.

### Phase 3: Supporting green care actors

To increase GreenME’s short- and long-term impact and the exploitation and dissemination of results from Phases 1 and 2, we began to build a tight-knit community for knowledge exchange, co-create green care national schemes, a European framework and cross-sectoral guidelines and create online training materials for green care actors. Throughout, the project is supported by a communications team which is working to further exploit the results of the project through various media outlets, webinars and workshops planned with the EU bubble.

#### Building a tight-knit community for knowledge exchange

GreenME is fostering a tight-knit green care community for knowledge exchange that will be the basis for a broader European Green Care Network. We first identified key actors that best support the development, implementation and sustainability of green care, starting with existing consortium contacts and the green care actors identified during Phase 1. The green care community is structured in national chapters, each with 10 to 15 participants and a chapter coordinator. Participants in each country chapter have been invited to a series of country-specific and international workshops and will receive information on activities organised over the course of the project.

#### Co-creation of green care national schemes and guidelines for scaling up green care

Responding to concern about the fragmentation of green care strategies and policies, country chapter members will co-create^36^ green care national schemes and guidelines for scaling-up green care to improve mental health equity. Each chapter is developing unique actionable measures for basic criteria and procedures that can be adapted by their countries and establish how different actors can better interact with each other to influence and improve the integration of green care across sectors. These actionable measures will also outline how to adapt and apply GreenME findings to other contexts, starting with the potential for knowledge transfer between countries. We will use a multi-stage methodology to co-create guidelines and national schemes combining formal and informal consensus approaches and expert discussions. Through a series of workshops during which participants will be presented with and discuss evidence from Phases 1 and 2, each country chapter will propose, discuss and validate measures across the three levels of green care to improve mental health and well-being equity while ensuring social and ecological co-benefits.

Two international workshops will be conducted, with the final joint workshop (online) focusing on knowledge transfer in a broader global north context, building on the interim results from country chapters and generating guidelines to be used in all European countries and a general European green care framework on how to integrate and advance green care. The guidelines will be cross-sectoral based on the national schemes developed by each country chapter and will align with European policies and plans such as the EU Green Deal and EU biodiversity strategy 2030.

#### Developing online learning resources for NBT providers

The key learning and process from each phase of the project will be transformed into a set of online learning resources designed to be user-led and non-linear to support the varying needs of organisations and practitioners working in the field of NBT and other aspects of green care in the Global North.

### Patient and public involvement

Our study includes as its target population both those who may be considered patients and members of the general population who may benefit from exposure to some type of green care (from NEL to nature-based therapies). As such, we employed a transdisciplinary approach, including partners who serve as practitioners, local government representatives and not only researchers. Phase 1 focuses on capturing the perspectives of these multiple disciplines and stakeholders on the subject of the status of green care. Phase 2 includes practitioner partners in the design and implementation of the studies. Meanwhile, in Phase 3, we include multiple stakeholders, including patient representative organisations in dissemination efforts.

### Project status

The GreenME project officially launched in September 2023. Ethical approval has been obtained for each phase of the project from all relevant ethics committees. Phase 1 data collection has been completed. For Phase 2, trials for evaluating the effectiveness, causal pathways and cost-effectiveness of NBT are underway and data collection for the assessing the mental health and well-being effects of NBP and NEL is complete and data is currently being analyzed. Each country has recruited members of their national chapters for Phase 3 and has held their first/introductory meetings.

### Ethics and dissemination

Ethical approval for the various phases of the project has been obtained from the following ethical boards: 1) Spain: Research Ethics Committee of the Universitat Autonoma de Barcelona, 6594 (General), 6851 (Phase 1), UAB-CERec180 (Phase 2), UAB-CERec178 (Phase 2) and CEEAH 7269 (Phase 3), (WP4, Phase 2 classified as exempt by the UAB); Ethics Committee of the Universitat Oberta de Catalunya, CE23-PR29 (General), CE240-PR07 (Phase 1), CE24-PR59 (Phase 2), CE24-PR55 (Phase 2), (WP4 of Phase 2 was considered exempt); Comitè d’Ètica d'investigació Clinica amb Medicaments de l'Hospital de Mataró, CEIm 11/25 (Phase 2); CEIm Fundació Sant Joan de Déu, PIC-82-25 (Phase 2); 2) Italy: Comitato di Bioetica of the Alma Mater Stadiorum Universita di Bologna, Proj no. 0157655 (Phase 1) and 0075975 (Phase 2); 3) Germany: ILS Ethics Review Panel, no number ‘Survey in Workpackage 2’ (Phase 1), no number ‘Workshops in Workpackage 5’ (Phase 3); 4) Sweden: Swedish Ethics Review Committee, 2024-2810-01 (Phase 1), 2025-0195401 (Phase 2); 5) UK: Central Research Ethics Advisory Group, University of Kent, CREAG064-04-024 (Phase 1); University of Salford Ethics Administration, Ref 0189 (Phase 1), 4552 (Phase 2); 6) Poland: Institute of Psychiatry and Neurology Bioethics Committee, no number (Phase 1); Committees on Ethics and Scientific Research, Res no 32/RKF/2023U (Phase 1).

Each trial from Phase 2 (1 or 2 in each country) has been registered separately in the ISRCTN registry. The following numbers correspond to the trials in the study: ISRCTN13105773, ISRCTN71485431, ISRCTN11841855, ISRCTN14970007 and ISRCTN15381820.

All study results will be disseminated through peer-reviewed publications, scientific conferences and via reports for policy-making bodies at the regional (European Union), national and local levels. Results will also be disseminated for the general public through the project website and via our multiple non-academic consortium partners.

## Discussion

The GreenME project aims to build evidence for the use of green care to improve adult mental health and well-being equity throughout the Global North, ultimately contributing to more just, resilient and sustainable communities. GreenME’s strengths lie in its transdisciplinary approach—leveraging partnerships with local community actors in each study country to generate results that respond to the actual concerns and limitations faced by practitioners and policy-makers.

The project addresses several gaps in the literature. First, it seeks to provide more robust evidence for the potential of green care at all levels (NEL, NBP, NBT) to improve mental health and well-being (equity). While many studies have evaluated nature-based therapies or other nature-based programmes, most are small-scale and focus on evaluating only one specific intervention case. Furthermore, our initial diagnosis of green care status in partner countries indicates that existing evidence is not yet sufficiently robust to advocate for the widespread acceptance and use of the more targeted aspects of green care (such as NBT and NBP) and their integration into formal healthcare systems. GreenME is also, to our knowledge, the first large-scale study to evaluate the effectiveness of green care from a health equity perspective, centring the needs and preferences of those who have the greatest needs for mental health and well-being support due to experiences of marginalisation. Here we expand existing evidence on inequities in access to green and blue spaces and begin to think about who may be lacking fair access to good quality, targeted nature-based therapies and programmes.

Our approach requires expertise from many sectors and disciplines, together forming a large, multi-cultural and transdisciplinary consortium to successfully employ the methods we propose here. Participants include academic researchers, local government administration representatives, providers of green care interventions and communication experts. In particular, the co-creation process requires extra efforts in mediating diverse goals and priorities expressed by green care implementers, decision-makers and academics. We have several administrative processes in place to bolster our ability to maintain contact and communications and ensure all consortium members reflect on their privileges (ie, holding regular check-ins with consortium partners, creating an agreed-upon list of common terms, opening participation in working groups to address more specific issues within the consortium to those who are interested and power and privilege reflective activities in each general assembly meeting).

However, GreenME’s approach has its challenges. Learning from existing interventions requires thoughtful consideration of the trade-offs between study design and practical feasibility. In evaluating existing NBTs, applying key features of randomised-control trials is often difficult and, in some cases, not possible (such as blinded randomisation, achieving sufficiently large sample sizes for cost-effectiveness analyses, standardising intervention features and others) while still respecting the integrity of the individual interventions. Nevertheless, much can be gained from this approach, particularly in building the evidence base needed to scale-up the use of NBT in the future and to develop long-term funding and operational frameworks that would otherwise remain beyond the scope of this study.

A further challenge concerns our focus on health (in)equity. We anticipate challenges in recruiting representative samples for all study phases, particularly to maintain a sufficient sample of marginalised groups. This is important given our overarching hypothesis that those marginalised groups who might be most in need of green care to support mental health and well-being may not be receiving the benefits of such care due to their marginalised status. Our consortium, including multiple countries, further compounds the challenges due to cultural and ethical differences concerning data collection on dimensions of marginalisation such as race, ethnicity, sex, gender and socioeconomic status. The project has thus formed an internal working group consisting of representatives from each project country to focus on standardising the methods for measuring and analysing marginalisation within each study phase.

The goal of the GreenME project is to produce new, robust and impactful evidence that will help improve how green care is delivered and accessed and enhance its mental health and well-being benefits for those who may need it most. GreenME thus has the potential to contribute to building more equitable, just, climate-resilient, sustainable and healthy environments, aligned with the priorities of the EU Biodiversity Strategy and the Sustainable Development Goals.

## Supplementary material

10.1136/bmjopen-2025-114034 online supplemental file 1

## Data Availability

No data are available.
