# Supplementary material for: Learning from real-world conditions to advance green care for mental health equity in the Global North: protocol for a transdisciplinary multi-scalar project
Source: BMJ Open. 2026 Jul 29;16(7):e114034. doi: 10.1136/bmjopen-2025-114034 (PMC13422925; doi:10.1136/bmjopen-2025-114034)
Supplement: online supplemental file 1 [file bmjopen-16-7-s001.docx]

## Annex 1

### Interview questions and focus group instructions

#### Semi-structured interviews guidelines

**Before interview:**

Introduce the researcher, the project, and the interview, which explains:

- Who you are,
- What is the research project about,
- Aims and objectives of interview, 40-60 mins long,
- Consent and confidentiality.

**Notes about the structure of questions below:**

- Actual questions are in green. You can adapt them, keeping the meaning of the question.
- Any text in black is explanatory, and You can use it as you like, but be mindful of time!
- The Roman numerals I and II stand for clarification questions formulated appropriately for use in conversation with the I – Policy and decision maker; II- Implementer, III - Supporter of green care for (if you wish to use them in this way).

Please start the interview with some background and comfort questions to put the interviewee at ease, get the conversation started and ascertain the interview metrics that we haven’t already got in the 2.1. database.

- *Hi, I am X and I represent X one of the partners within the GreenME project*
- *Did you read / understand the background information that you were sent about this interview and the GreenME project before the interview?*
- *Do you have any questions?*
- *Are you happy for this interview to go ahead (i.e., consent)?*
- *I will be recording this interview so that it can be transcribed and analysed are you ok with this? (your responses will be anonymised)*
- *The most important principle concerns the full freedom to express your opinions. There are no right or wrong answers, just different perspectives!*

**Intro** (to explain or even read at loud to our interviewee):

*GreenME: Advancing GReenCare in Europe: an integrated multi-scale approach to the expansion of nature-based therapies to improve mental health equity is an international project funded by the European Commission (type of the project: HORIZON-CL6-2022-COMMUNITIES-02-02) and joints universities, NGO’s and other participants of seven countries (Spain, Poland, Sweden, Italy, Germany, Great Britain, United States of America) around green care research and promotion.*

*GreenME understands green care as a three-scale continuum from nature-in-everyday-life (e.g. the existence of green and blue infrastructure for viewing and walks which benefit people’s health) to nature-based health promotion (the promotion of active interaction with nature such as gardening and conservation activities that end up benefiting people’s health) to nature-based therapy (the provision of treatment for individual patients). How do you understand the relationship between nature and mental health or mental well-being?*

Timing 6 mins max

**Q1 – How do you find the state of green care in your country/region? Do you think the 3 levels of green care model is present in your country/region?**

**Tag STATUS**

*Are these 3 levels as understood as providing health benefits for different population groups? Which groups?*

*I Is 'green care' organized according to a viable economic model? Are the three levels integrated and coordinated?*

*II How does it affect your work on green care? What particularly helps you with your daily practice?*

*III What do You think about therapies with nature, environmental health promotion, contact with nature in daily life in your country?*

Timing 6 mins max

**Q2 - What types of natural environments are used for green care delivery? Why?**

*I, II, III How do You think of managing existing greenery (parks, urban parks, allotments and community gardens, forest, urban woodlands and so on) to increase health benefits? Why?*

**Tag AREAS CHARACTERISTICS**

Timing 5 mins max

**Q3 - Related to this, do you know of any recognised best practice models, protocols, or guidelines for delivering ‘nature-based therapy’ in your country/region? Are these used specifically in relation to mental health? (i.e., in the treatment of mental ill health or the promotion of wellbeing for example)**.

**Tag BEST PRACTICES**

*I What would be the ideal scenario for 'green care' in your region/country? How do you think it could be introduced on a wider scale as part of the primary healthcare system?*

*II When implementing green care, what can you say about procedures involving contact with elements of nature offered by public healthcare or private providers? Are these cases and models known?*

*III How do You find green care in your site? What benefits you can see?*

Timing 8 mins max

**Q4 - Are you aware of guidelines and rules according the green care operating in your country?**

**Tag PROVISION GUIDELINES**

*I, II, III Do you know any standards related to the presence and operation of green care? Regulations that affect the operation of such care providers? Are there any standards that you must adhere to?*

Timing 5 mins max

**Q5 - Are there established nature-based health promotion tools or protocols in your country? Do you use them?**

**Tag PROMOTION TOOLS**

*II This may include guidelines for the promotion of the use of green and blue spaces for mental health and well-being purposes to the public. Official or non-official channels and types of information available to the public on the function of green and blue spaces, as well as nature-based health promotion programs? Existing urban and community gardens promoting healthy activities.*

*Do You believe or not if the population at your country is involved in promoting green care? Do You know any associations/institutions coming from the civil society involved in this?*

Timing 7 mins max

In the middle of the interview, it's worth asking another 'ice-breaking' question, e.g. 'Thank you, I've already learned a lot from you. Let's talk for a moment more about....

**Q6 - How are greenery and the natural environment used to prevent, promote and enhance mental well-being in your region/country?**

**Tag PROMOTION ACTIVITIES**

*I How it refers to local policies communications/education? How is this promoted for marginalised groups?*

*II What mental health problems/diseases do these prevention/promotion activities focus on?*

*III How can you use the greenery in your daily life? How do You know about?*

Timing 6 mins max

**Q7 - What barriers do you see to contact with nature being used in your team to prevent, promote and treat adults with mental illness?**

**Tag BARRIERS&CONFLICTS**

*I What barriers do you see to contact with nature being used in your team to prevent, promote and treat adults with mental illness? Please think about financial, technical, legislative, management, recognition and integration barriers. How do you manage referrals to ensure that the people they support are appropriate for the service provision?*

*II What barrier particularly bothers you in your daily work?*

*III Can You see any barriers in green care you can see in you day-to-day environment?*

Timing 6 mins max

**Q8 - Do you believe that all residents in your area have equal possibilities to benefit from nature-in-everyday life, nature-based-promotion and nature-based-therapies?**

*I, II, III Do all social groups have same access to nature-in-everyday life, nature-based-promotion and nature-based-therapies? And in terms of use, are there any differences? Why? If not, what groups benefit more and why? And what benefit less and why? Are there any interventions or policies in place to decrease these differences in benefit?? (Please note i.e. marginalised residents such as immigrants, racial/ethnic minorities, elderly, women, etc.).*

**Tag EQUAL ACCESS**

Timing 6 mins max

**Q9 - What other green care stakeholders do you work with? Would it be important for you to cooperate with any entities? Which ones?**

**Tag COOPERATION**

*I, II, III With which organizations or individuals do you have interactions with respect to the three levels of green care (nature-in-everyday-life, nature-based health promotion, nature- based therapy)***.** *In what capacity could you cooperate? What specific contributions could you make? With which entities and institutions would you like to collaborate?* *Are there barriers? Could we learn from partnership working in this field in other countries?*

Timing 5 mins max

#### Focus group interviews

We recommend to provide focus groups interviews after semi-structured interviews and grey literature review as experts’ interviews to validate and verify results, deepen answers and check for possible contradictions in the statements.

You can use the same questions as in the semi-structured interview, or, regarding above add new questions if needed.

### Tagset Codebook

**TAGSET AREAS CHARACTERISTIC**

- **TAG ACH_ Area Types** – types of greenery or other places - parks, gardens, allotment gardens, community gardens and other urban gardens, hospital gardens, at health centres, nursing homes, senior homes, others
- **TAG ACH_ Areas Description** – description of existing green spaces (public and private areas) and other areas - features of the sites, their appearance, key elements determining their suitability for green care; solutions used in them
- **TAG ACH_Desirable Areas** - description of desirable green (or other) areas - descriptions of imagined or green spaces seen abroad (public and private areas) changes in the area management to increase health benefits
- **TAG ACH_Facilities** – description of facilities providing green care, accessibility equipment
- **TAG ACH_Other** – other content to be encoded

**TAGSET BARRIERS & CONFLICTS**

- **TAG B&C_Context** – system-related issues e.g. referral mechanisms, unfavourable policy environment, lack of buy-in from healthcare etc.
- **TAG B&C_Funding** – financial issues e.g. amount, timing/duration of funding, restrictions, sources.
- **TAG TAG B&C_Logistical** – practical issues e.g. access, size, transport, natural space limitations, facilities etc.
- **TAG B&C_Capacity** – adequate staff skills for patient need, training, project resources, demand, time, numbers - staff to participant ratios etc.
- **TAG B&C _Other** – other content to be encoded.

**TAGSET BEST PRACTICES**

- **TAG BP_Practical** - practice and delivery related e.g. shared learning and expertise, tools, practical ‘how to’ guides etc.
- **TAG BP_Collaborations** – successful ways of working with other stakeholders e.g. via local and national networks or working partnerships with other organisations
- **TAG BP_Funding** – financing e.g. sustainable, transferrable or scalable funding/ models
- **TAG BP_Management** – governance, practices, and management structures e.g. planning, monitoring and evaluation
- **TAG BP_Other** – other content to be encoded

**TAGSET COOPERATION**

- **TAG COO_Potential -** potential for future cooperation; any statement about possible cooperation partners
- **TAG COO_Existing** - existing cooperations any information about current & past cooperation partners **TAG COO_Problems -** barriers to cooperation: problems that may have hindered the formation of a cooperation, aspects which need to be improved to establish more cooperation
- **TAG COO_Goals-** goals of the cooperation: aspects that explain or show what the cooperation is/was about**;** possible benefits the cooperation created reasons, why the cooperation came to be**;** any reference made to possible contents or goals of future cooperations
- **TAG COO_Other** – other content to be encoded

**TAGSET EQUITY_JUSTICE** (previous EQUAL_ACCESS)

- **TAG EQJ_Equitable Access -** equal/equitable (or not!) access to nature-in-everyday- life, nature-based promotion and nature-based-therapies; who and why has more or less access; equity practices for improving access (e.g. free transport for those low income to access nature outside the city, free or lower prices of green therapy for marginalised communities^[[1]](#footnote-1)^), other examples of equal/equitable (or not) access and barriers or opportunities influencing on equitable access.
- **TAG EQJ_Equitable Benefits -** benefits (especially mental health benefits) from exposure to contact with nature being the same for everybody (or not), those that need it the most (marginalised communities^1^) benefitting more from nature (or not); reasons for different (mental) health benefits from nature gained by different population groups.
- **TAG EQJ_Policies Interventions -** other aspects e.g.: the possibility of participation and influence decisions is similar/differential between groups, any interventions or policies in place to: (i) improve access to nature-in-everyday-life, nature-based promotion and nature-based-therapies; (ii) to ensure access to - and benefit from- by those who need it the most; (iii) equality, equity or justice aspects included in health or environmental plans.
- **TAG EQJ_Target Groups** – groups considered for green interventions; community groups mentioned by the interviewee, reasons for these groups to be the target, the way these groups are considered in nature-health interventions/policies/practices.
- **TAG EQJ_Other** – other content to be encoded, e.g. historical injustices/inequities.

**TAGSET PROMOTION_ACTIVITY**

- **TAG PRA_Health_Outcome:** mental health problems mentioned; health improvements expected or sought; causes of health through nature promotion.
- **TAG PRA_PARTICIPANTS** - main targets and participants of these activities; modes of action adapted or directed at them.
- **TAG PRA_Problems** – problems and barriers to promote and enhance mental health.
- **TAG PRA_Inequities** – promotion and adaptation of the promotion for unprivileged communities^1^; social groups excluded from activities to promote mental health through green and blue spaces.
- **TAG PRA_Other** – other content to be encoded

**TAGSET PROMOTION_TOOL**

- **TAG PT_Level:** National, Regional, Local How do these activities appear in the local communication or education policies?
- **TAG PT_Channels:** Official or Non-official channels, How are the activities promoted to the population? Through which channels?
- **TAG PT_Media:** What activity is being used to promote mental health and wellbeing? Film, Book, Seminar, Homepage, Lecture, Free public lecture, Conference, Educational program, other Program
- **TAG PT_Initiative of promotion:** Professional (top-down); unprofessional (bottom-up)
- **TAG PT_Other** – other content to be encoded

**TAGSET PROVISION GUIDELINES**

- **TAG PG_Environmental Regulations** - national and local environmental laws and regulations; biodiversity conservation; water resource management; pollution reduction
- **TAG PG_Health and Welfare Regulations** - quality of services offered; staff training; safety and hygiene procedures; specific regulations for social cooperatives
- **TAG PG_Employment and Training Regulations** - training requirements for green care operators; skills necessary to work with nature
- **TAG PG_ Financial and Management Regulations** - financial reporting; obtaining public funding; fund management

**TAGSET STATUS**

- **TAG ST_Understanding -** way understanding the three levels of green care in relation to different target groups
- **TAG ST_Organization -** organization of the green care, viable economic model, integration and coordination of the three levels of green care, the scale of green care actions
- **TAG ST_Everyday Work** – daily activity by green care actor, factors affecting work on green care; aids, opportunities and obstacles to everyday work
- **TAG ST_Daily Reality** – reality of therapies with nature, environmental health promotion and contact with nature in daily life.
- **TAG ST_Other – other content to be encoded**

### The consent form

**Sample declaration of consent to participate in a scientific study and consent to the processing of personal data in connection with participation in a scientific study**

I, the undersigned, .......................................................................................................... declare that I have read and understood the above information regarding the described scientific study entitled Assessment of the current state of green care as part of the project entitled Development of green therapies in Europe: An integrated multi-scalar approach to the development of nature-based therapies to improve mental health justice (Acronym GreenMe, project number 101084198, program type: Horizon Europe), including research conducted in this field, I had the opportunity to asking questions and I received comprehensive and satisfactory answers to my questions. I voluntarily consent to participate in this research study and am aware of the fact that I may withdraw my consent to participate in the further part of the research study at any time without giving a reason.

☐ YES

☐ NO

Name and surname of the participant in the scientific research project

………………………………………………………………………………………....................................

Signature…………………………………………..

Place and date……………………………………

By signing the consent to participate in a scientific study, I do not waive any of my rights. I will receive one of two copies of this form, signed and dated.

By signing this document, I also confirm that I have been informed about the method of processing data from the scientific study and that this data is collected only for the purpose of scientific analysis of the study.

Name and surname of the participant in the scientific research project

………………………………………………………………………………………....................................

Signature…………………………………………..

Place and date……………………………………

### The self reporting questionnaire for GreenMe respondents

Please provide us with some data about the environment of your work and the position you held. The GreenME team will use this information only for scientific purposes. Participation in the survey is anonymous.

1. What entity do you represent?

- Municipality
- Ministry
- NGO
- University
- healthcare institution
- other

2. Headquarters of the entity:

- large city: over 100 thousand of inhabitants
- medium town: 20-100 thousand of inhabitants
- small town: less than 20 thousand inhabitants
- village
- other

3. What position do you held?

- head of department
- departmental director
- company owner
- employee
- other

4. What is your substantive scope of responsibilities?

- administration
- spatial planning
- environmental protection
- provision of health services
- social care
- nature conservation
- research
- education and training
- insurance
- finance
- consulting
- tourism
- media
- other

5. Name of the administrative unit

6. Length of professional experience

- up to 5 years
- 5-10 years
- 10-20 years
- over 20 years

7. What is your professional background

Thank you for answering!

1. Determinants of mental health vary significantly for **communities facing structural disadvantages or discrimination resulting in having fewer resources or living in physical or social conditions which are less conducive to promoting good mental health than others**. Examples of such groups may inclue older adults, racially or ethnically marginalised populations, populations with low socioeconomic status (SES), women and non-cisgender individuals, and immigrant communities, among others, depending on the social context (hereafter referred to as underprivileged communities”)(Mezzina et al., 2022.) [↑](#footnote-ref-1)
