# Supplementary material for: Learning from real-world conditions to advance green care for mental health equity in the Global North: protocol for a transdisciplinary multi-scalar project
Source: BMJ Open. 2026 Jul 29;16(7):e114034. doi: 10.1136/bmjopen-2025-114034 (PMC13422925; doi:10.1136/bmjopen-2025-114034)
Supplement: Supplementary file 2 [file bmjopen-2025-114034.reviewer_comments.pdf]

PEER REVIEW HISTORY

BMJ Open publishes all reviews undertaken for accepted manuscripts. Reviewers are asked to complete a checklist review form and are provided with free text boxes to elaborate on their assessment. These free text comments are reproduced below.

ARTICLE DETAILS

Title (Provisional)

Learning from real-world conditions to advance Green Care for mental health equity in the Global North: Protocol for a transdisciplinary multi-scalar project

Authors

Cole, Helen; Caputo, Silvio; Specht, Kathrin; Giedych, Renata; Otsuka, Noriko; Cook, Penny A; Gawryszewska, Beata; Luciano, Juan V.; Olszewska-Guizzo, Agnieszka; Hedblom, Marcus; Houston-Read, Rebecca; McVay, Katelyn; Neuberger, Konrad; Sienkiewica-Jarosz, Halina; Feliu-Soler, Albert; de Prado-Bert, Paula; Hardman, Michael; Howarth, Michelle Louise; Bragg, Rachel; Crichigno, Antonella; Triguero-Mas, Margarita

---

VERSION 1 - REVIEW

**Reviewer**	**1**
**Name**	**Wilson, Rebecca**
**Affiliation**	**University of Bristol, NIHR ARC West**
**Date**	**13-Apr-2026**
**COI**	**None**

This is a thorough and comprehensive protocol and I learned a lot reading it. I just have a couple of comments, mostly minor, for clarification.

Introduction

Really interesting and comprehensive introduction, I enjoyed reading it. I have just a couple of suggestions for clarity.

Line 15 - Can you include a brief description of the marginalised groups, rather than refer the reader to Table 1

Line 25 – Can you please define blue spaces, this isn't a term I have heard before

Lines 39-50 – This paragraph is really interesting, could you say very briefly why marginalised individuals might benefit more from exposure to nature? The mechanism isn't obvious to a naïve reader

Methods

The examples in the definitions table are really helpful, they are a helpful way of actually understanding some of these more left-field ideas

Whilst there may be populations of marginalised groups within, all included countries are high income countries in the 'global north', are the limitations of this discussed anywhere? The social deprivation is relative only to the country and does not compare with the global south. I'd just be careful not to overstate the socioeconomic diversity of the program.

Why was only grey literature reviewed? This would exclude a large body of evidence and does require some justification.

The "analysis of grey literature" is very vague, could you describe some search methods? And how results were synthesised?

Discussion

Line 15 – I believe 'Fdto' is a typo

VERSION 1 - AUTHOR RESPONSE

Reviewer: 1
Dr. Rebecca Wilson, University of Bristol
Comments to the Author:

This is a thorough and comprehensive protocol and I learned a lot reading it. I just have a couple of comments, mostly minor, for clarification.
Response: Thank you! And thank you for your time and helpful comments to improve the paper.

Introduction

Really interesting and comprehensive introduction, I enjoyed reading it. I have just a couple of suggestions for clarity.

Comment: Line 15 - Can you include a brief description of the marginalised groups, rather than refer the reader to Table 1
Response: We have added a brief description in the text. Thank you for pointing this out.

Comment: Line 25 – Can you please define blue spaces, this isn't a term I have heard before
Response: We have added a brief description which we hope improves the understanding.

Comment: Lines 39-50 – This paragraph is really interesting, could you say very briefly why marginalised individuals might benefit more from exposure to nature? The mechanism isn't obvious to a naïve reader
Response: Thanks for pointing this out. This is indeed an interesting point, and there are many theories about which this pattern is seen in past analyses on the relationship between exposure to nature and health. Some include that margnalised groups often experience worse initial health outcomes, and thus the impact of exposure to nature may be more "visible" among these groups. Another is that marginalised groups may have worse access to other healthful resources, and therefore the role of nature in producing better health may be stronger than for other groups. We have added a brief mention of these justifications in the introduction section.

Methods

Comments: The examples in the definitions table are really helpful, they are a helpful way of actually understanding some of these more left-field ideas
Response: Thank you. We are glad you found it useful!

Comment: Whilst there may be populations of marginalised groups within, all included countries are high income countries in the 'global north', are the limitations of this discussed anywhere? The social deprivation is relative only to the country and does not compare with the global south. I'd just be careful not to overstate the socioeconomic diversity of the program.
Response: Yes, we certainly agree. Thank you for pointing this out. We have added a bit more nuance to the description in the text. This is something we discussed a lot about internally during our planning phases.

Comment: Why was only grey literature reviewed? This would exclude a large body of evidence and does require some justification.
Response: We employed grey literature in the evaluation of the state of green care in each study country/study area because we were specifically interested in how green care is being implemented (or not) in the study areas where we were working, as well as what sorts of laws, policies guidelines, or other supporting factors exist in these areas. Thus, we wanted to analyse the existing local, regional or local level documents which would allow us to understand this better. We have now expanded the description of the grey literature included and how it was analysed. We hope this better explains and justifies the use of grey literature. The analysis of the status of green care was also informed by semi-structured interviews with key actors in each study area.

Comment: The "analysis of grey literature" is very vague, could you describe some search methods? And how results were synthesised?

Response: We have included a new paragraph to explain this process, for which we used the READ approach. We realize also that while we mentioned the use of the READ approach, it was not clear that this was linked to the analysis of the grey literature. We hope the new descriptive paragraph is more clear.

Discussion

Comment: Line 15 – I believe ‘Fdto’ is a typo
Response: Thank you for catching this. It has been corrected.
